# Association of small intestinal bacterial overgrowth with nonalcoholic fatty liver disease in children: A meta-analysis

**DOI:** 10.1371/journal.pone.0260479

**Published:** 2021-12-02

**Authors:** Linghan Kuang, Wei Zhou, Yongmei Jiang

**Affiliations:** 1 Department of Laboratory Medicine, West China Second University Hospital, Sichuan University, Chengdu, China; 2 Key Laboratory of Birth Defects and Related Diseases of Women and Children (Sichuan University), Ministry of Education, Chengdu, China; McMaster University, CANADA

## Abstract

It has been suggested that small intestinal bacterial overgrowth (SIBO) could cause nonalcoholic fatty liver disease (NAFLD), but this association was not examined in children by meta-analysis. This meta-analysis aimed to determine the association between SIBO and NAFLD in children. The electronic databases PubMed, Embase, and Cochrane Library were searched for studies published before April 22, 2021. The outcome was the association between SIBO and NAFLD. Three studies and 205 children were included. All three studies reported the association between SIBO and NAFLD. Children with SIBO were more likely to have NAFLD (odds ratio = 5.27, 95% confidence interval (CI): 1.66–16.68, P<0.001; I^2^ = 63.5%, P_heterogeneity_ = 0.065). When directly pooling the reported relative risks (RR) from two studies, children with NAFLD had an over 2-fold increased relative risk of developing SIBO (RR = 2.17, 05%CI: 1.66–2.82, P<0.001; I^2^ = 0.0%, P_heterogeneity_ = 0.837). This meta-analysis reports a possible association between SIBO and NAFLD in children.

## Introduction

Nonalcoholic fatty liver disease (NAFLD) results from pathophysiological fatty liver changes unrelated to alcohol intake [1]. Histologic progression from steatosis to nonalcoholic steatohepatitis (NASH), fibrosis, and cirrhosis does not always correlate with clinical progression, especially in the early stages [1]. The causes of NAFLD include insulin resistance, obesity, weight gain, and diabetes [2]. The worldwide prevalence of obesity is 5.6% in girls and 7.8% in boys of 5–19 years of age, but the prevalence is >20% in many countries [3]. In addition, the prevalence of NAFLD is 7.6% in children [4].

The small intestinal bacterial overgrowth (SIBO) syndrome is a nutrient malabsorption condition associated with an excessive number of bacteria in the proximal small intestine [5, 6]. SIBO is often associated with bloating, pain, gas, and diarrhea [5, 6]. Predisposing factors for developing the SIBO syndrome include disorders of the protective antibacterial mechanisms, anatomic disorders such as blind loop or short bowel syndromes, and motility disorders [7]. The symptoms can be secondary to an underlying disease or due to the effects of toxic bacterial metabolites or nutritional complications such as fat malabsorption and vitamin deficiencies [7]. Its prevalence is unknown because of the non-specific symptoms of variable intensity (sometimes asymptomatic) that overlap with other diseases such as irritable bowel syndrome and inflammatory bowel disease [7].

According to recent epidemiologic studies, SIBO could be another risk factor for NAFLD [8–17]. The exact mechanisms linking SIBO and NAFLD are unknown but could involve increased intestinal permeability and bacterial toxins. Those toxins can trigger a low-grade inflammatory state that promotes insulin resistance and NAFLD [18–20]. In addition, endogenous ethanol production by the microbiota could predispose to NAFLD through lipid accumulation in the liver, oxidative stress, and gastrointestinal abnormalities allowing the passage of toxins through the gut liver axis [21, 22]. The gut microbiota can also cause choline deficiency, and choline is necessary for the secretion of very-low-density lipoproteins (VLDL), leading to triglyceride accumulation in the liver [23–26].

A recent meta-analysis showed that there was a significant association between NAFLD and SIBO [27], but this meta-analysis included studies of patients of all age groups. Considering the epidemics of obesity in children and the prevalence of NAFLD in children, examining the association between NAFLD and SIBO in this age group is clinically relevant, especially in the context where SIBO is a manageable condition [5, 6, 28]. Therefore, the present meta-analysis aimed to determine the association between SIBO and NAFLD in children.

## Material and methods

### Literature search

This meta-analysis was performed in accordance with the Preferred Reporting Items for Systematic Reviews and Meta-Analyses (PRISMA) reporting guidelines [29]. Since no original clinical raw data was collected or utilized, ethical approval was not required for this meta-analysis.

The eligibility criteria were 1) patients: children with obesity/overweight, 2) exposure: SIBO, 3) outcome: NAFLD, 4) study design: no limitation, and 5) full text published in English. The electronic databases PubMed, Embase, and Cochrane Library were searched for studies published before April 22, 2021, using the MeSH terms “Pediatric Obesity” and “small intestinal bacterial overgrowth” combined with relevant key words (S1 File). The reference lists of the retrieved articles were searched for additional potentially eligible studies.

### Data extraction and quality assessment

Potentially relevant publications were screened and evaluated independently by two reviewers (LH,K and W,Z), with a third reviewer (YM,J) resolving any disagreement and confirming data extraction. A structured data collection sheet was developed. Two researchers independently extracted data, including authors, year of publication, country, study design, sample size, age, percentage of males, and the diagnostic criteria of NAFLD and SIBO. The observational studies were evaluated according to the Newcastle-Ottawa scale (NOS) [30]. For cohort studies, the NOS evaluates the representativeness of the exposed cohort (2 points), the selection of the non-exposed cohort (1 point), the ascertainment of exposure (2 points), the demonstration that the outcome of interest was not present at the start of the study (1 point), the comparability of cohorts on the basis of the design or analysis (2 points), the assessment of the outcome (2 points), whether follow-up long enough for outcomes to occur (1 point), and the adequacy of follow up of cohorts (2 points). For case-control studies, the NOS evaluates the adequacy of the case definition (2 points), the representativeness of the cases (1 point), the selection of controls (2 points), the definition of controls (1 point), the comparability of cases and controls on the basis of the design or analysis (2 points), the ascertainment of intervention (2 points), the same method of ascertainment for cases and controls (1 point), and the non-response rate (2 points). The maximum score is 9 points. A study is considered of good quality when having 3–4 points in the selection domain (items 1–4) and 1–2 points in the comparability domain (item 5), and 2–3 stars in the outcome/exposure domain (items 6–8) [30].

### Statistical analysis

All analyses were performed using STATA MP 14.0 (StataCorp, College Station, Texas, USA). Odds ratios (ORs) with 95% confidence intervals (CIs) were combined for statistical analysis. Statistical heterogeneity among studies was calculated using Cochran’s Q-test and the I^2^ index. An I^2^ >50% and a Q-test P<0.10 indicated high heterogeneity, and the random-effects model was used; otherwise, the fixed-effects model was applied. P-values <0.05 were considered statistically different. Potential publication bias was not assessed due to too few studies being included [31].

## Results

### Selection of the studies

**Fig 1:**
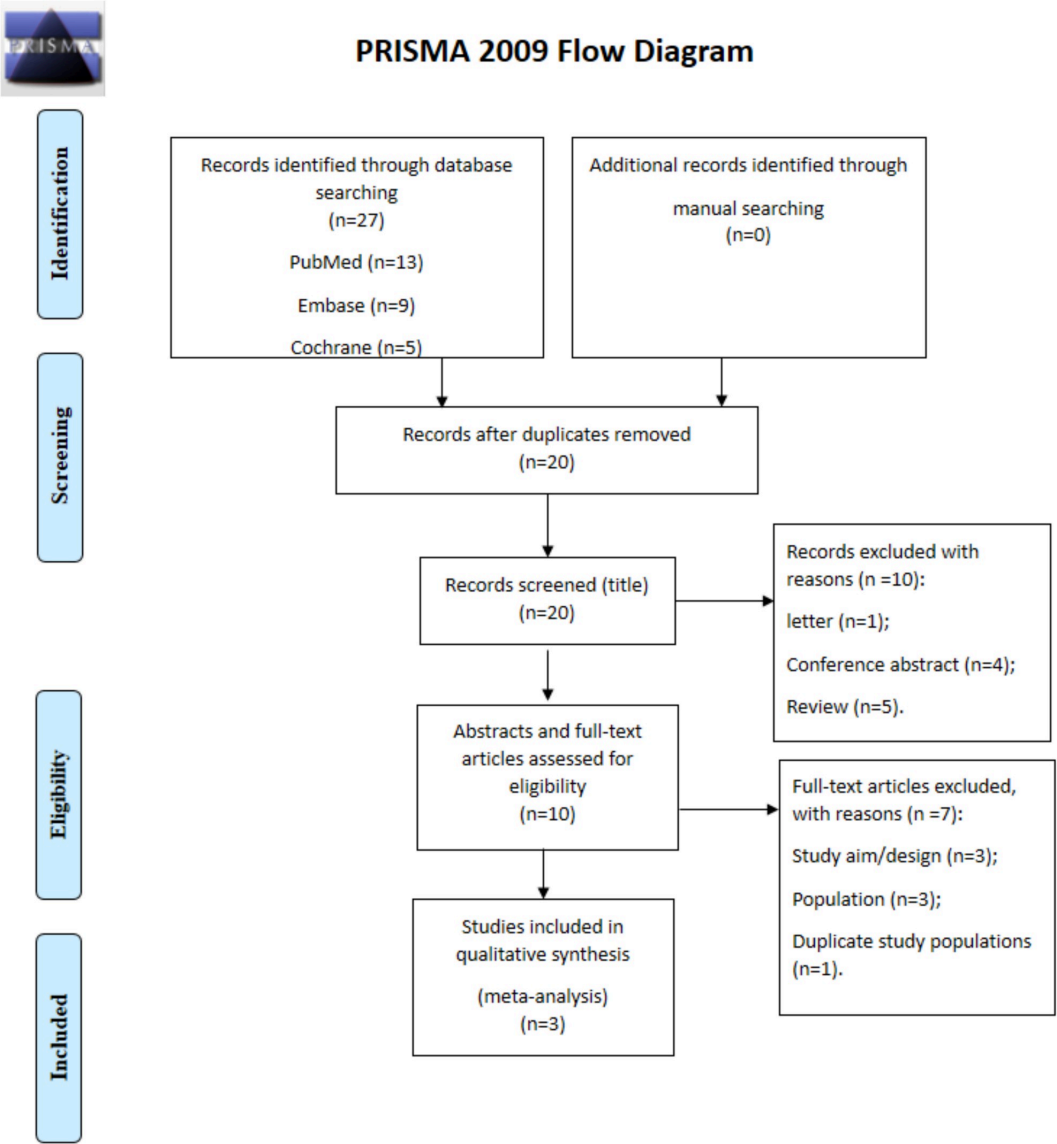
Study selection process.

Twenty-seven records were retrieved from the databases (PubMed, n = 13; Embase, n = 9), and 20 records were left after removing the duplicates. Ten records were excluded after assessing the titles (one was a letter, four were conference abstracts, and five were reviews), and ten full-text papers were assessed. Seven were excluded because of study aim/design (n = 3; the objective was not related to the present meta-analysis), population (n = 3; those studies only included adults, not children), and because of duplicate study populations (n = 1) (Fig 1).

Finally, three studies were included [32–34] (Table 1). The three studies included 205 children. The mean age ranged 10.8–14.2 years, and 62.4% were boys. There were two case-control studies [32, 34] and one cohort study [33]. The cohort study [33] scored 7 on the NOS, and the two case-control studies [32, 34] scored 8 on the NOS (S1 Table).

**Table 1:** Characteristics of the included studies.

Author, Year	Troisi, 2017 [32]	Belei, 2017 [33]	Stepanov, 2019 [34]
**Country**	Italy	Romania	Ukraine
**Study type**	Case-control study	Cohort study	Case-control study
**Sample size (n)**	22	125	58
**Without SIBO (n)**	10	78	28
**SIBO (n)**	12	47	30
**Without SIBO age (years)**	11.6±2.1 (not reported according to SIBO)	14.2 ± 2.2	10.8±2.8
**SIBO age (years)**		15.5 ± 2.4	11.8±2.7
**Without SIBO F/M**	9/13	27/51	11/17
**SIBO F/M**		18/29	12/18
**Without SIBO BMI**	27.6±4.6 (not reported according to SIBO)	27.4 ± 3.1	24.4±3.8
**SIBO BMI**		27.9 ± 3.1	24.4±3.8
**Diagnostic criterion of NAFLD**	Children with ultrasonographic bright liver ± hypertransaminasemia underwent transaminase retesting, creatine phosphokinase determination, and laboratory exclusion of the most frequent causes of pediatric liver disease other than NAFLD (autoimmune hepatitis, Wilson disease, celiac disease, alpha1-antitrypsin deficiency, viral hepatitis A, B, and C, Cytomegalovirus, and Epstein Barr virus).	The diagnosis of NAFLD was made according to the European Society for Pediatric Gastroenterology, Hepatology and Nutrition (ESPGHAN) and the North American Society of Pediatric Gastroenterology, Hepatology, and Nutrition (NASPGHAN) guidelines based on abdominal imaging methods.	Diagnosis of NAFLD was established according to CAP and exclusion of secondary steatosis in children with overweight/obesity.
**Diagnostic criteria of SIBO**	Small intestinal bacterial overgrowth was identified using a hydrogen breath test (H2BT) apparatus. H2 basal values >40 ppm or an increase of 20 ppm over baseline within the first 120 min were considered suggestive of SIBO.	The diagnosis of SIBO was based on a positive GHBT.	The diagnosis of SIBO was determined from GHBT and data using the Gastro+Gastrolyzer gas analyzer.
**Inclusion/exclusion criteria**	The inclusion criteria were 1) age between 5 and 16 years, 2) normal weight (BMI from the 25th to 85th percentile), 3) obese (BMI >95th percentile), and 4) absence of acute intercurrent or chronic illness.	The inclusion criteria were 1) consecutive overweight and obese children and adolescents, and 2) aged 10–18 years old with BMI between the 85th and 95th percentile and >95th percentile, respectively.	/
		The exclusion criteria were 1) children diagnosed with different liver disorders caused by other conditions than NAFLD (infectious hepatitis, autoimmune hepatitis, drug-induced liver injuries, Wilson’s disease, hemochromatosis, celiac disease, mucoviscidosis, alpha1-antitrypsin deficiency) or 2) adolescents with a history of alcohol intake.	

SIBO: small intestinal bacterial overgrowth; NAFLD: nonalcoholic fatty liver disease; GHBT: glucose hydrogen breath test.

### Association between SIBO and NAFLD in children

**Fig 2:**
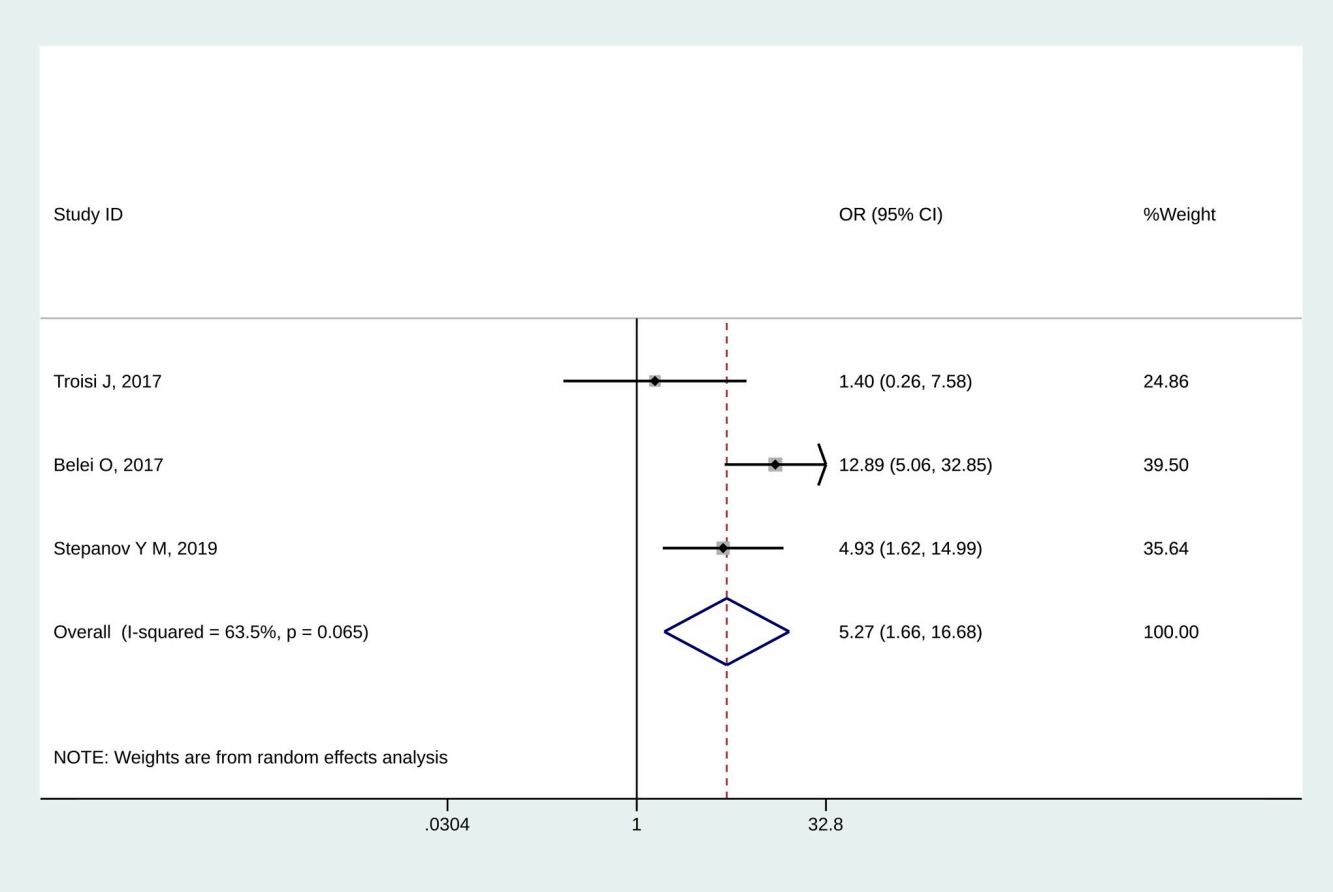
Forest plot of NAFLD.

All three studies [32–34] reported the association between SIBO and NAFLD. Children with SIBO were more likely to have NAFLD (OR = 5.27, 95%CI: 1.66–16.68, P = 0.005) (Fig 2). Moderate heterogeneity was observed (I^2^ = 63.5%, P_heterogeneity_ = 0.065).

### Risk of SIBO in children with NAFLD

**Fig 3:**
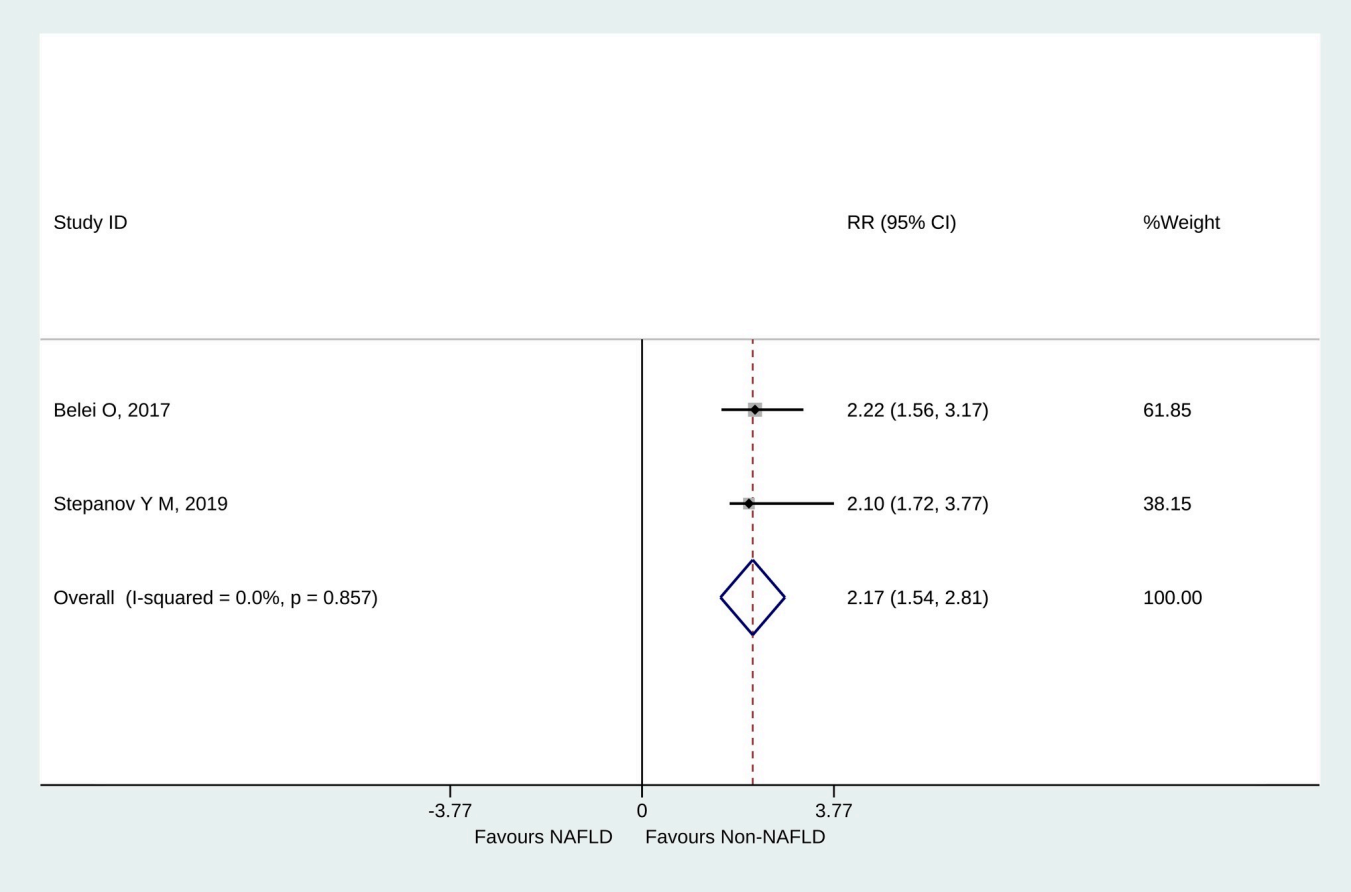
Forest plot of NAFLD (pooled from RR and 95%CI directly).

When directly pooling the reported relative risks (RR) from two studies [33, 34], children with NAFLD had over 2-fold increased relative risk of developing SIBO (RR = 2.17, 05%CI: 1.54–2.81, P<0.001) (Fig 3). No heterogeneity was observed (I^2^ = 0.0%, P_heterogeneity_ = 0.857).

### Sensitivity analysis

**Fig 4:**
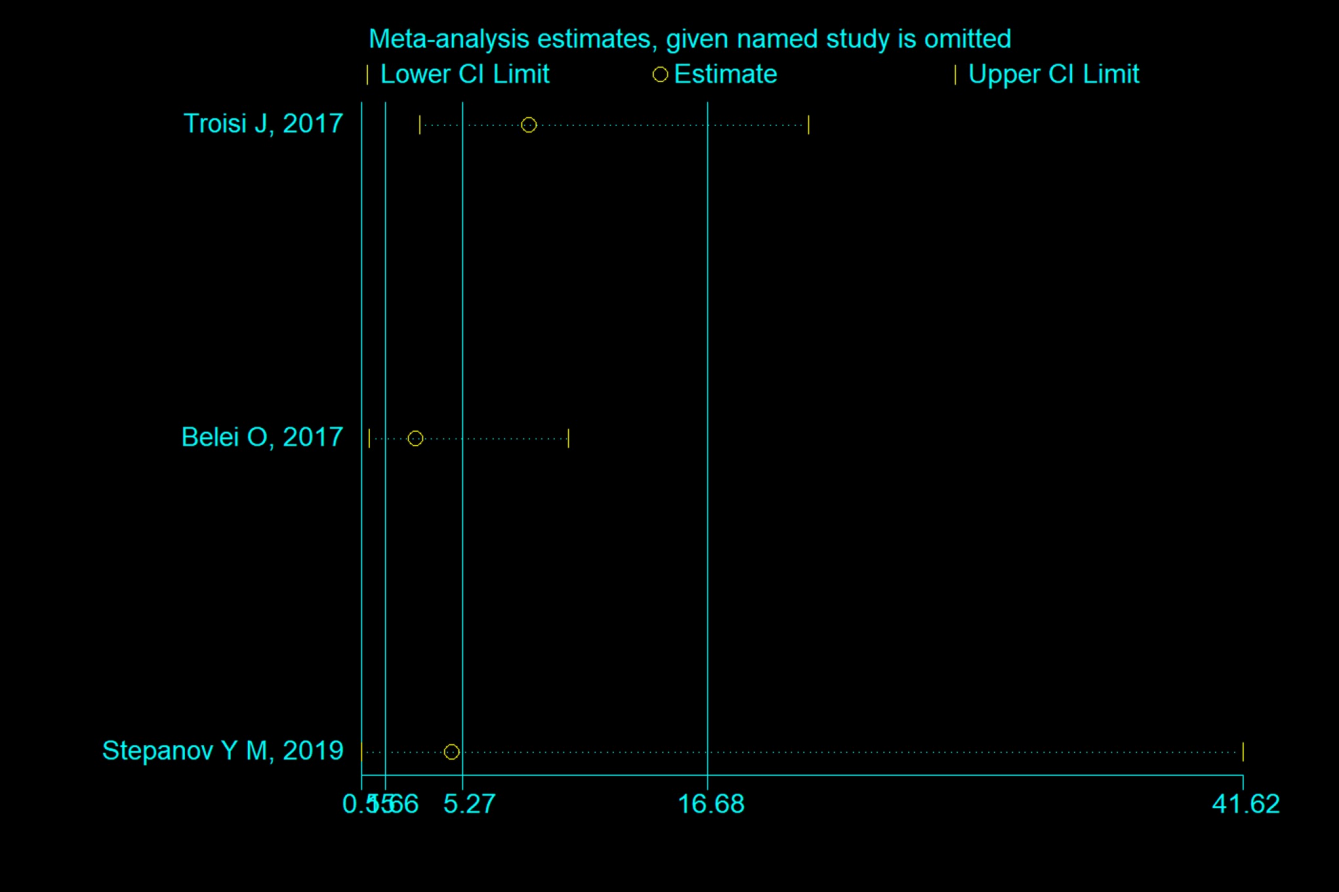
Sensitivity analysis of NAFLD.

The sensitivity analysis indicated that none of the three studies influenced the outcome (Fig 4).

## Discussion

It has been suggested that SIBO could be a cause of NAFLD [8–17], but this association was not examined in children by meta-analysis. Therefore, this meta-analysis was performed to determine the association between SIBO and NAFLD in children. The results indicate a significant association between SIBO and NAFLD in children. This is supported by a meta-analysis of ten studies, irrespective of patients’ age [27].

Biologically, the explanation for this association is currently unknown. Nevertheless, some hypotheses are being explored. Intestinal leakage might be a major culprit because of the dysregulated tight junctions observed in SIBO [11, 35]. Impaired tight junctions will allow bacterial products such as endotoxins (including lipopolysaccharides (LPS) and unmethylated DNA sequences) to enter the circulation, causing a low-grade systemic inflammatory reaction [36]. Low-grade endotoxemia in SIBO will activate the TLR-4 and CD14 receptors, which will activate the NF-κB pathway and lead to the expression of inflammatory cytokines [8, 36]. These inflammatory cytokines can directly participate in the pathogenesis of NASH [37], insulin resistance, and hepatic fibrosis [8, 38–40]. The chronic inflammatory state can lead to dysregulated lipid storage in the adipose tissue and insulin resistance [41, 42].

Other possible mechanisms for the development of NAFLD in SIBO include the production of ethanol as a bacterial metabolic byproduct, leading to the hepatic accumulation of triglycerides and oxidative stress, and mucosal abnormalities that facilitate the entry of toxins into the organism [21, 22]. The intestinal bacteria also produce short-chain fatty acids (SCFAs) that will reduce intestinal motility, increase the absorption of bacterial products [43] and participate in dysregulated glucose metabolism [40, 44, 45]. In addition, the overpopulation of bacteria in the small intestine will lead to the catabolism of choline by the bacteria, preventing its absorption. Choline is required for the secretion of VLDL particles by the liver, and impaired VLDL secretion will prevent the liver from exporting exogenous and endogenous lipids and will lead to hepatic lipid accumulation, steatosis, and NAFLD [23–26].

In fact, the mechanisms of SIBO are poorly understood. The pathogenesis of SIBO includes disorders of the protective antimicrobial mechanisms (achlorhydria, pancreatic exocrine insufficiency, and immunodeficiency syndromes), anatomical abnormalities (stricture, diverticula, fistulae, surgical blind loop, and previous ileocecal resections), motility disorders (secondary to systemic sclerosis, autonomic neuropathy, postradiation enteropathy, and small intestinal pseudoobstruction), and the long-term use of proton pump inhibitors [7]. In addition, SIBO involves multiple strains of bacteria, and the exact proportions might vary among individuals [46]. Still, which mechanisms are responsible for SIBO specifically in children is unknown. In addition, the causes of SIBO in the included studies were not reported. Furthermore, SIBO is a heterogeneous condition with variable clinical presentation and variable microorganisms involved [5–7], resulting in different rates of progression of NAFLD in different patients. The traditional model of NAFLD development is the two-hit model, but current evidence and hypotheses suggest that it is likely that the various mechanisms involved in NAFLD, including SIBO, participate in parallel to the development of NAFLD but with different proportions of involvement in different individuals [36, 47, 48]. Additional studies are necessary to examine the causes and mechanisms of SIBO in the pathogenesis of NAFLD.

The management of SIBO involves the treatment of the underlying diseases or abnormalities, eradicating bacterial overgrowth, and maintenance of remission [6, 7, 28], and correcting vitamin B12 deficiency and other associated nutritional deficiencies [6, 7, 28]. The options for eradicating bacterial overgrowth (induction of remission) include antibiotics to selectively target bacterial strains causing SIBO syndrome if possible (e.g., rifaximin 1200 or 1600 mg/day for 7–10 days as one treatment course or as cyclic therapy [49]; other options include tetracycline, neomycin, norfloxacin, amoxicillin/clavulanate, or erythromycin) [6, 7, 28]. Other treatment options, but with limited evidence, include a 14-day elemental diet, which is reported to normalize lactulose breath tests in patients with irritable bowel syndrome and abnormal lactose breath test, use of prokinetics for motility-associated SIBO syndrome, and probiotic therapies [6, 7, 28, 50]. The strategies for maintenance of remission include watchful observation and the use of promotility medication [6, 7, 28]. Still, the antibiotics used in the management of SIBO, especially repeated exposure, can have a potential impact on NAFLD. Studies showed that alterations in the gut microbiota drive the progression of NAFLD and that targeting the gut microbiota using probiotics [51] or antibiotics [52, 53] can slow the progression of NAFLD. Therefore, the relationship between SIBO and NAFLD is probably affected by the treatments undertaken in the patients. Furthermore, there is a risk of liver injury in patients with repeated antibiotic exposure [54–56] that could mimic the inflammatory changes observed in NAFLD [57]. Unfortunately, a major limitation of the present meta-analysis is that the included studies present very limited information about past treatments in their patients. Therefore, this issue remains unsolved and will have to be examined in future studies.

A meta-analysis is limited by the included studies. The study by Troisi et al. [32] was designed to analyze the urinary metabolomic of gut liver axis abnormalities. They diagnosed SIBO using a hydrogen breath test (H2BT) apparatus. H2 basal values >40 ppm or an increase of 20 ppm over the baseline within the first 120 min were considered suggestive of SIBO. The study by Belei et al. [33] directly examined the association between SIBO and the risk of developing NAFLD in children. SIBO was diagnosed using the glucose hydrogen test. In Stepanov et al. [34], the association between SIBO and obesity and overweight was examined in children. The diagnosis of SIBO was determined using the glucose hydrogen breath test and Gastro+ Gastrolyzer gas analyzer. Although all studies used hydrogen production to diagnose SIBO, different methods have different sensitivity and specificity [58, 59]. Still, SIBO can be caused by non-hydrogen-producing bacteria, and such tests would yield false-negative results [58, 59]. Future studies should use reliable and comprehensive diagnostic methods for SIBO.

Nevertheless, SIBO is a manageable condition [5, 6, 28]. Since the present study reported an association between SIBO and NAFLD, managing SIBO might be a target to prevent NAFLD development and progression in children. Developing NAFLD during childhood carries a potentially worst prognosis than developing it during adulthood, and managing NAFLD as early as possible should yield a better prognosis [60–62]. The world is currently seeing epidemics of childhood obesity [63]. Screening for and managing SIBO, especially in obese children, could be a way to prevent childhood NAFLD. Indeed, although NAFLD is multifactorial and depends upon BMI [4], not all obese children will develop NAFLD, and some non-obese children can also develop NAFLD. Since SIBO is preventable, it could be targeted to prevent NAFLD and its morbidity in adulthood. The use of antibiotics, prebiotics, probiotics, and synbiotics could be explored in the future [64–66].

This meta-analysis has limitations. Only observational studies were included, and their sample size was generally small, resulting in a small number of patients that could be included. The estimates were non-adjusted, preventing the accurate determination of the ORs and RRs. The studies used different diagnostic methods. Some of those methods are known to be less reliable than others [58, 67], and it is possible that some studies missed some children who should have been included. The use of different diagnostic methods, different study designs, and different study populations resulted in heterogeneity, which could affect the conclusions. It was also unclear whether the criteria for SIBO were the same among the four studies, especially for the study by Belei et al. [33]. Obesity is a strong confounder of NAFLD, and it is not clear if SIBO alone affects both obesity and NAFLD or SIBO affects obesity resulting in NAFLD development. Unfortunately, the small number of studies and patients included in this meta-analysis prevented the inclusion of only normal-weight children with SIBO. Most of all, the present meta-analysis highlights the lack of data regarding the association between SIBO and NAFLD in children.

## Conclusions

In conclusion, the present meta-analysis determined that there might be an association between NAFLD and SIBO in children. Importantly, this meta-analysis highlights the lack of data available about SIBO and NAFLD in children. Still, this meta-analysis is hypothesis-generating and highlights the lack of data available in children on this topic. Future studies should explore the mechanisms linking SIBO and NAFLD and examine whether treating SIBO could prevent NAFLD in children. Future trials should be designed with the aim of exploring the causality relationship between SIBO and NAFLD and improving the diagnosis of SIBO. Standardized diagnostic criteria should be formulated for SIBO by an authoritative organization.

## Supporting information

S1 Checklist PRISMA 2009 checklist. (DOC)

S1 Table Quality assessment of included studies based on NOS. (DOCX)

S1 File Electronic search strategy of PubMed. (DOCX)
