# Supplementary material for: Association of small intestinal bacterial overgrowth with nonalcoholic fatty liver disease in children: A meta-analysis
Source: PLoS One. 2021 Dec 2;16(12):e0260479. doi: 10.1371/journal.pone.0260479 (PMC8638857; doi:10.1371/journal.pone.0260479)
Supplement: S1 Table — (DOCX) [file pone.0260479.s002.docx]

**S1 Table.** Quality assessment of included studies based on NOS

| NOS cohort | Representativeness of the exposed cohort | Selection of the non exposed cohort | Ascertainment of exposure | Demonstration that the outcome of interest was not present at the start of the study | Comparability of cohorts on the basis of the design or analysis | Assessment of outcome | Was follow-up long enough for outcomes to occur | Adequacy of follow up of cohorts | Total quality scores |
| --- | --- | --- | --- | --- | --- | --- | --- | --- | --- |
| Belei, 2017^33^ | ☆ | ☆ | ☆ | - | ☆☆ | ☆ | - | ☆ | 7 |
| NOS case-control  Study | Is the case definition adequate? | Representativeness of the cases | Selection of controls | Definition of controls | Comparability of cases and controls on the basis of the design or analysis | Ascertainment of intervention | Same method of ascertainment for cases and controls | Non-response rate | Total quality scores |
| Troisi, 2017 ^32^ | ☆ | ☆ | - | ☆ | ☆☆ | ☆ | ☆ | ☆ | 8 |
| Stepanov, 2019 ^34^ | ☆ | ☆ | - | ☆ | ☆☆ | ☆ | ☆ | ☆ | 8 |
