# Supplementary material for: Association of small intestinal bacterial overgrowth with nonalcoholic fatty liver disease in children: A meta-analysis
Source: PLoS One. 2021 Dec 2;16(12):e0260479. doi: 10.1371/journal.pone.0260479 (PMC8638857; doi:10.1371/journal.pone.0260479)
Supplement: S1 File — (DOCX) [file pone.0260479.s003.docx]

Electronic search strategy

Pubmed

#1 (("Pediatric Obesity"[Mesh]) OR ((Obesity, Pediatric) OR (Childhood Onset Obesity) OR (Obesity, Childhood Onset) OR (Obesity in Childhood) OR (Child Obesity) OR (Obesity, Child) OR (Childhood Obesity) OR (Obesity, Childhood) OR (Infant Overweight) OR (Overweight, Infant) OR (Infantile Obesity) OR (Obesity, Infantile) OR (Infant Obesity) OR (Obesity, Infant) OR (Childhood Overweight) OR (Overweight, Childhood) OR (Adolescent Overweight) OR (Overweight, Adolescent) OR (Adolescent Obesity) OR (Obesity, Adolescent) OR (Obesity in Adolescence))) 93685

# 2 small intestinal bacterial overgrowth 1856

#3 #1 AND #2 13
